# Alveolar ridge reconstruction with titanium meshes: A systematic review of the literature

**DOI:** 10.4317/medoral.19998

**Published:** 2014-09-30

**Authors:** Marco Rasia dal Polo, Pier P. Poli, Davide Rancitelli, Mario Beretta, Carlo Maiorana

**Affiliations:** 1 Marco Rasia dal Polo 1, Pier-Paolo Poli 2, Davide Rancitelli 3, DDS. Clinical assistant professor, Department of Dental Implants. U.O.C. Maxillofacial Surgery and Odontostomatology. Fondazione Cà Granda IRCCS. University of Milan, Milan, Italy; 2 DDS. Resident, Department of Dental Implants. U.O.C. Maxillofacial Surgery and Odontostomatology. Fondazione Cà Granda IRCCS. University of Milan, Milan, Italy; 3 DDS, PhD Fellow. Department of Dental Implants. U.O.C. Maxillofacial Surgery and Odontostomatology. Fondazione Cà Granda IRCCS. University of Milan, Milan, Italy; 4 DDS, PhD. Clinical assistant professor, Department of Dental Implants. U.O.C. Maxillofacial Surgery and Odontostomatology. Fondazione Cà Granda IRCCS. University of Milan, Milan, Italy; 5 MD, DDS. Professor and Chairman, Department of Oral Surgery and Dental Implants. U.O.C. Maxillofacial Surgery and Odontostomatology. Fondazione Cà Granda IRCCS. University of Milan, Milan, Italy

## Abstract

Alveolar bone regeneration by means of titanium meshes is a widespread procedure, however to date, only few relevant studies were reported in literature concerning this technique. Consequently, the aim of the present systematic review was to analyze the reliability of the titanium mesh as a barrier, in conjunction with horizontal and vertical ridge reconstruction for implant placement purposes. A total of 17 articles complying with the inclusion and exclusion criteria were reviewed. Three outcome variables were defined: a) horizontal and vertical bone regeneration obtained, b) complication rate, defined as the percentage of membrane exposures and c) evaluation of implant survival, success and failure rate.In regards to the vertical regeneration the mean was 4.91 mm (range: 2.56 - 8.6), while a mean of 4.36 mm (range: 3.75 - 5.65) was calculated for horizontal reconstruction. Considering the exposure rate, a mean of 16.1% was found, nevertheless, implant placement were placed in almost all of the sites. A mean success rate of 89,9%, a mean survival rate of 100% and a failure rate of 0% emerged from the data evaluation. A meta-analysis could not be performed due to the heterogeneity of the data, however the final results were comparable with those reported in case of bone regeneration obtained through other types of non-resorbable membranes. An advantage in favour of the titanium mesh was found in terms of bone loss after exposure, as implant placement was not jeopardized in almost all of the cases. It could be deduced that titanium meshes represented a reliable solution for alveolar ridge reconstruction. The clinical studies currently available in literature have shown the predictability of this technique in both lateral and vertical bone regeneration.

** Key words:**Alveolar ridge reconstruction, bone atrophy, bone regeneration, dental implants, titanium mesh.

## Introduction

The promising developments from the osteointegration field, the biomaterials and the surgical techniques have made the implant prosthetic rehabilitation a routinely approach during the treatment of partially and totally edentulous patients. However, especially in case of long lasting edentulous ridges, the residual bone volume is often not satisfying to place dental implants in a prosthetic driven procedure, as requested by the most recent aesthetic guidelines. The reestablishment of an adequate amount of bone and a proper contour of the alveolar ridge, has consequently become mandatory to allow a prosthetically driven implant placement. Se-veral surgical techniques have been therefore developed to augment the residual bone volume, including guided bone regeneration (GBR) with resorbable or non-resorbable membranes (1). GBR biological rationale is based on the mechanical exclusion of undesirable soft tissue cells from growing into the osseous defects, allowing only osteogenic cell populations derived from the parent bone to repopulate the osseous wound space (2). This is favoured by the use of barrier membranes, which can create a secluded space over the area to be augmented, in order to stabilize the blood clot and to exclude the soft tissue penetration. The protected space is then colonised by osteogenic cell populations resulting in new bone formation (3,4). Materials used as a barrier must be characterized by some physico-chemical characteristics, to provide for biocompatibility, tissue integration, cell occlusivity, space-making ability and clinical handling (5). Consequently, barrier membranes had been grouped as resorbable or non-resorbable. Non-resorbable barriers include expanded (e-) or high density- (d-) polytetrafluroethylene (PTFE) and titanium meshes (6). Considering the latter being part of GBR devices, it could be controversial, due to the fact that the impermeability towards competing non-osteogenic soft tissue cells is missing, nevertheless, the use of titanium during volumetric alveolar bone increasing procedures prior to implant placement is still on-going and seems to be a predictable solution in both horizontal and vertical augmentation (6). Such statement could be interpreted as a consequence of the several biomechanical properties belonging to this material. Porous titanium meshes through excellent space-making properties, could act as a containment system for the particulate bone or bone substitutes placed beneath the membrane, thus preventing the soft tissues collapse and the resulting compression or displacement of the graft during the entire healing period. The handling is anyhow maintained, so that clinicians could bend, contour and adapt the titanium mesh to the bone defect, miming the tridimensional architecture of the desired alveolar ridge outline (7). Another clinical advantage is the lower rate of exposure of the titanium mesh in comparison with e-PTFE membranes (8). Furthermore, titanium mesh when exposed might not have to be immediately removed, as the presence of pores allows a proper vascular supply to the underlying tissues, without interfering with the blood flow (7). As data concerning the present technique were lacking, the objective of this systematic review, based on current publications, was to evaluate the reliability of the titanium mesh as a device applied to horizontal and vertical bone augmentation procedures, for dental implant prosthetic rehabilitation purposes.

## Material and Methods

-Search strategy

A literature search based on PubMed, MEDLINE, EMBASE and ScienceDirect databases was conducted using the following research keywords: a) “guided bone regeneration titanium mesh”; b) “oral titanium mesh”; c) “vertical bone augmentation titanium mesh”; d) “vertical ridge augmentation titanium mesh”. The initial search included manuscripts from 1973 up to 2013 in which bone augmentation procedures with titanium mesh were performed and was complemented by an additional manual search, analysing the most relevant article bibliographies on the following topic-related dental journals (Impact Factor range: 1.197-3.961): *Clinical Oral Implant Research, International Journal of Oral and Maxillofacial Implants, Journal of Oral and Maxillofacial Surgery, International Journal of Periodontics and Restorative Dentistry, Journal of Cranio-Maxillofacial Surgery, Journal of Clinical Periodontology, Journal of Periodontology, Periodontology 2000, the British Journal of Oral and Maxillofacial Surgery, Clinical Implant Dentistry and Related Research, International Journal of Oral and Maxillofacial Surgery, The Journal of Oral Implantology*. Two reviewers performed the literature search independently.

-Inclusion and exclusion criteria

The inclusion and exclusion criteria were selected at the beginning of the study. The literature search was limited to dental journals and restricted to articles published in English language. Only manuscripts based on in vivo protocols, including at least 5 patients per study, wherein implants were inserted and functionalized from at least 6 months were reviewed, in order to evaluate any possible biological complications during the function, rather than early failures. Randomized and non-randomized clinical studies, cohort studies, case-control studies and case series have been considered, while case-reports have been excluded. Studies reporting not the entirely outcome measurements, but providing information on the augmentation procedure amount, were also included. Studies involving patients with heavy smoker habits (>20 cigarettes/day), and/or previously affected by tumours or congenital malformations have been dropped out. Regarding researchers from the same group, the final publication was selected after having found a continuity relation with the previous studies. Implants success rate was evaluated following Albrektsson *et al.* criteria consisting in: absence of persistent subjective complaints such as pain, foreign body sensation and/or dysaesthesia; absence of mobility; absence of peri-implant radiolucency and infection with pus suppuration and marginal bone resorption (MBR) not exceeding 1.5 mm after the first year of loading and up to 0.2 mm yearly thereafter (9). Implants respecting those criteria presenting a higher MBR were considered survived.

-Outcome variables

The following three outcome variables were defined: a) horizontal and vertical bone regeneration obtained, b) complication rate defined as the percentage of membrane exposures, and c) evaluation of implant survival, success and failure rate according to Albrektsson *et al.* criteria (9).

-Data extraction

Two reviewers screened the data independently using extraction tables. Any disagreements were resolved by discussion aiming for consensus. The screening process included three phases. Initially, all headlines were checked so as to exclude irrelevant manuscripts or animal and in vitro studies. Afterwards, publication abstracts were screened in order to analyse the type of surgery, patients’ number and characteristics. Finally, remaining publications complying with the inclusion/exclusion criteria listed above, were included in the present review. A meta-analysis of the data reported in the papers included in the present review could not be performed, due to the heterogeneity of the data within a similar group of clinical situations. As a consequence, it was not possible to perform an assessment of the risk of bias within the studies.

## Results

**Figure 1:**
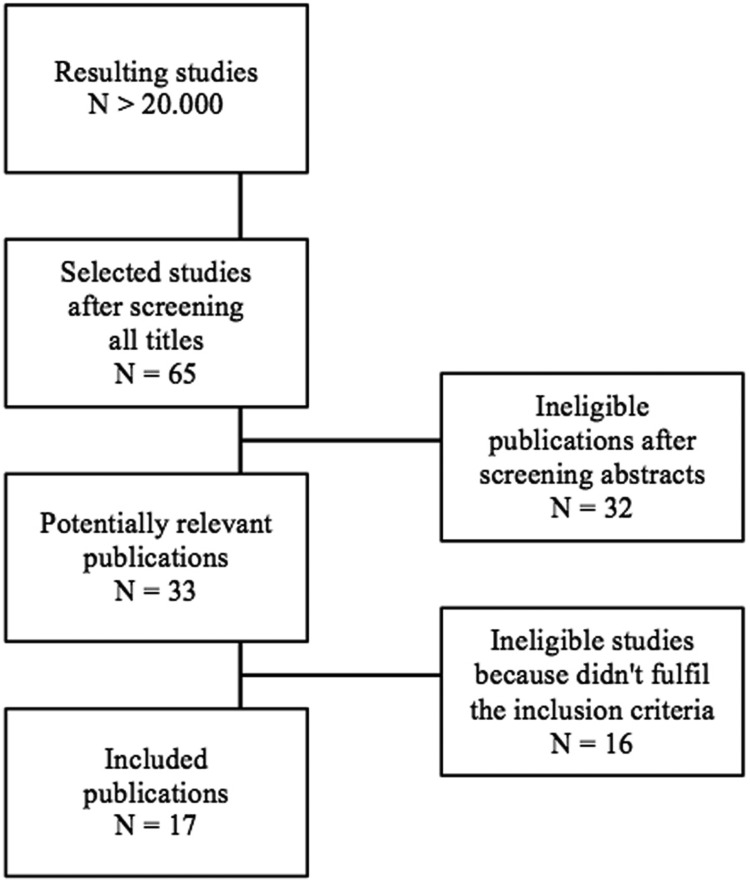
List of publications remained after full text analysis and subsequently reviewed.

Approximately 20.000 titles resulted from the systematic electronic search,ranging from 1973 up to 2013. The initial screening and evaluation of these headlines led to a reduction of 65 titles. Subsequently, 33 potentially relevant manuscripts were included after abstracts review. A total of 16 articles were excluded after an accurate evaluation comparing inclusion and exclusion criteria, associated with the reason for exclusion. Finally, 17 publications remained after full text analysis and were than reviewed (Fig. 1).

Due to the heterogeneity of the data acquired, results highlighted the lack of a consistent number of articles related to the surgical protocol investigated. Regarding the total of articles included (n=17), 10 were case series, 3 were prospective studies, 2 were retrospective studies and 2 were cohort studies. During the evaluation of cohort studies, only data related to patients who received titanium mesh were reported. The number of patients recruited in the selected studies was > 10 among all articles except one (10), resulting in a mean of 17,38 patients (range: 5-30). Both maxillary and mandible were treated in almost all of the studies, with the exception of Gongloff *et al.* (11) and Malchiodi *et al.* (12) who regenerated only upper jaw atrophies, and Misch *et al.* (10) who considered only lower jaws. The number of treated sites reached an average of 18,5. Most of the studies associated vertical and horizontal bone augmentation (13/17). Concerning the grafting material used, autogenous bone both in a block or in a particulate form, was the preferred material (13/17), eventually combined with other biomaterials. Deproteinized bovine bone mineral (DBBM) was used in 6 out of 17 studies, alone or generally associated with autograft. If combined, bone: DBBM concentration ratio was reported in only 3 articles: Maiorana *et al.* (13) and Proussaefs *et al.* (14) used a 50:50 ratio, while Pieri et al. (15) grafted a 70:30 ratio. The combination of acellular collagen, allografts and bone morphogenetic proteins (BMP) was mentioned in one study (10), however data regarding the success of this technique could not be extrapolated. Finally, only 2 studies (16,17) preferred the blood clot without any grafting material, waiting no more than 6 months to re-entry. Thus, the healing period remained comparable to the rest of the reviewed studies, indeed the overall mean healing time was 5.9 months. Analysing the above-mentioned timespan, two different healing treatment options have emerged, a long waiting protocol (8-9 months) versus a short one (3-4 months). The tendency to consider two different healing times depending on the treated jaw seemed clear, approximately a mean of 6 months for the upper maxilla and 4 months for the mandible.

a) Evaluation of the Horizontal/Vertical bone regeneration obtained

Clinical data related to the volumetric measurements inherent to the newly formed bone amount achieved using titanium mesh were reported in  Table 1. Values of the obtained bone regeneration amount were reported in 10 out of 17 articles. When analysing the number of surgical sites the mean was 17.7, while 19.5 was the mean value concerning the number of patients. In regards to the vertical regeneration the mean was 4.91 mm (range: 2.56 - 8.6), while a mean of 4.36 mm (range: 3.75 - 5.65) was calculated for horizontal augmentation. Proussaefs *et al.* reported the lowest regeneration measurements (2.56 mm for the vertical and 3.75 mm for the horizontal augmentation) associated with one of the highest percentage of mesh exposure (35,2%), presuming a possible cause-effect relation (14). On the other hand, with a rate of exposure close to the former (33.3%), Roccuzzo *et al.* obtained a vertical bone regeneration of 4.8 mm that could be considered on the average of the other articles (18). It have to be pointed out that Leghissa *et al.* achieved the highest vertical regeneration without any grafting material, only separating epithelial cells from the blood clot, reaching 8.9 mm (16). The mean healing period elapsed before removing the titanium mesh was approximately 7 months, ranging from 3.5 months (16) to 9 months (19). The mean vertical augmentation was 8.6 mm and 5.2 mm respectively. Concerning the grafting materials used, particulated autogenous bone graft was predominant, followed by DBBM alone or combined with autologous bone graft. Onlay autogenous bone block grafts were used in two studies, whereas in only one previously reported case, no filling materials were used.

**Table 1:**
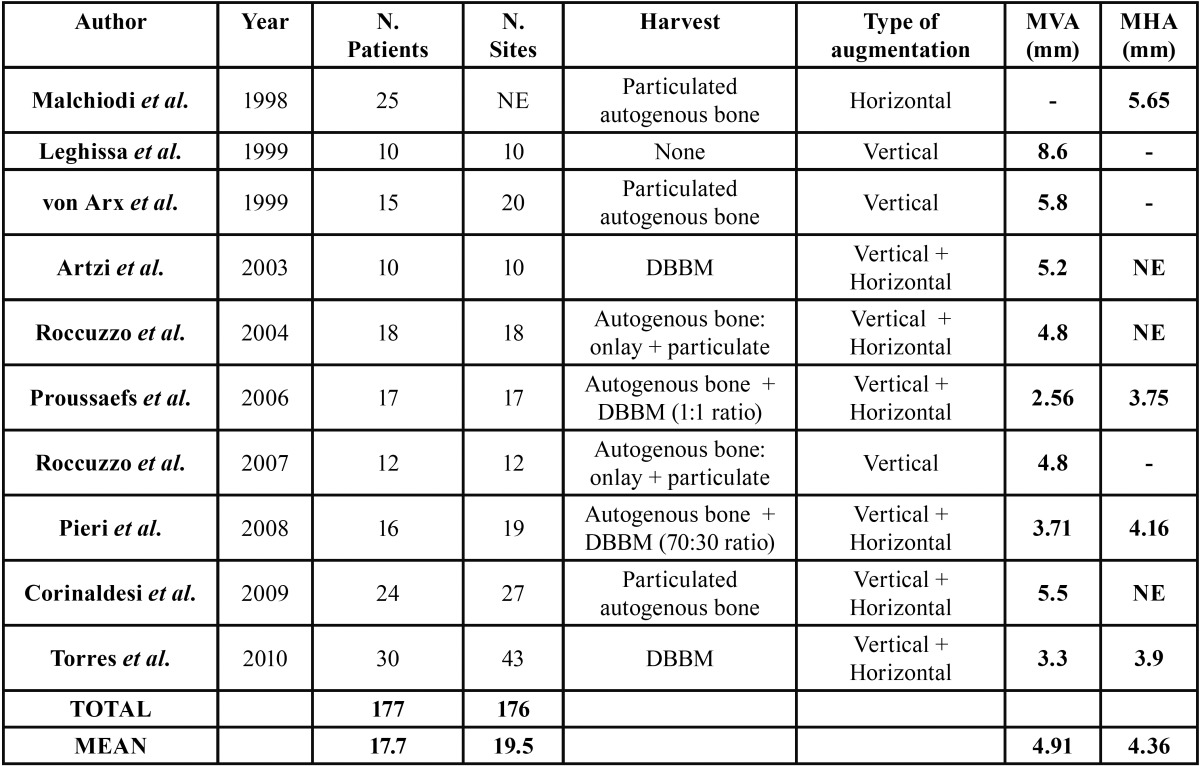
Evaluation of the Horizontal/Vertical bone regeneration. MVA: Mean Vertical Augmentation; MHA: Mean Horizontal Augmentation; NE: Not Evaluable; DBBM: Demineralized bovine bone mineral.

b) Evaluation of GBR related complications

Clinical data related to bone regeneration complications were reported in  Table 2. The exposure of the meshes was the most frequent complication in this type of surgery. The mean emerged exposure rate was 16.1%. TiMe technique described by von Arx *et al.* (20) reached the major rate of exposure (50%), while no complications occurred in 3 studies. In most cases, the exposure was not followed by titanium mesh removal, however a reduction of the bone gain was observed in half of the studies; nevertheless implant insertion was not possible in only one case, meaning that the exposure did not jeopardize the result in almost all of cases.

**Table 2:**
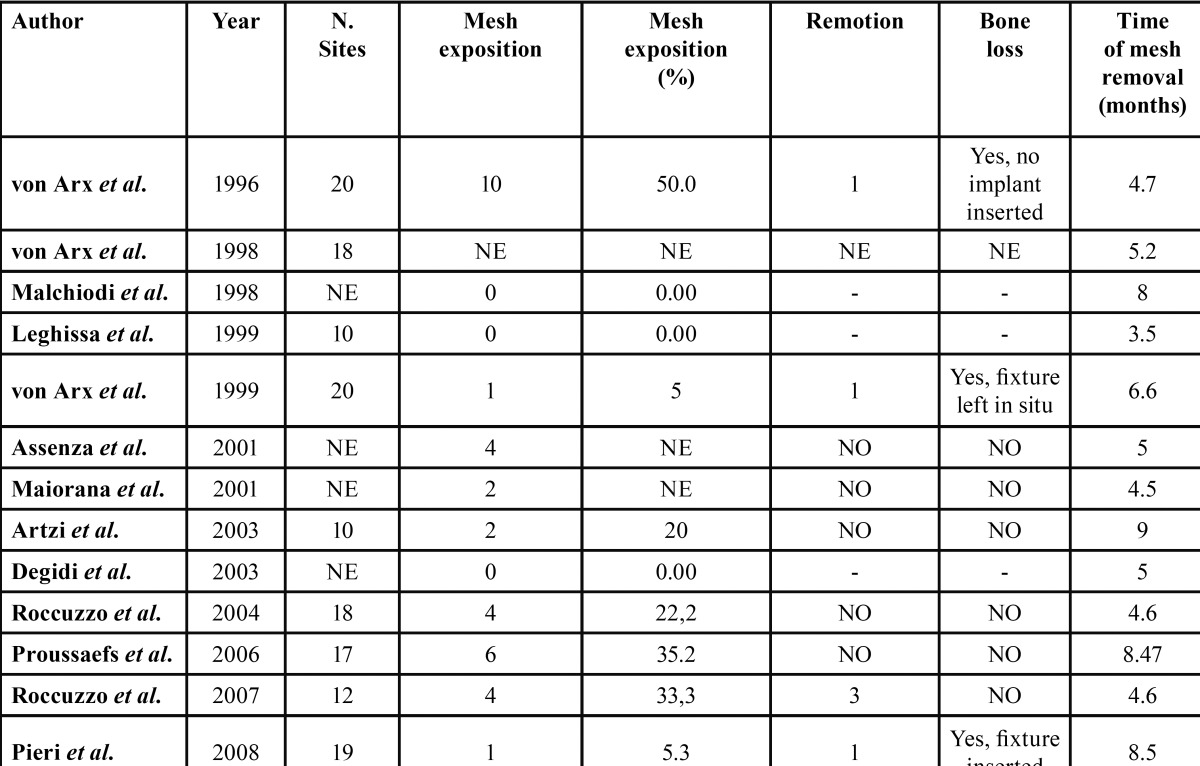
Evaluation of titanium mesh exposure. NE: Not Evaluable.

c) Evaluation of implant survival, success and failure rate 

Implant success rate was evaluated considering at least 6 months from the prosthetic load. Clinical criteria assumed for the evaluation of the success rate were those proposed by Albrektsson *et al.* previously described (9). Implants fulfilling previous criteria yet presenting a higher MBR, were considered survived. Considering the entirety of the articles screened, a total of 645 dental implants were placed in 324 patients. Regrettably, few studies have adopted the above-mentioned guidelines in reporting implants related data, hence final results were limited ( Table 3). The mean abutment connection timespan was 3 months and during the consecutive follow-up starting from the six months, merely 7 implants were lost. Overall, four studies re-entered in the previously proposed criteria for the implants assessment. Of the 130 analysed implants, the success rate was 89,9% with 0% failures. Accordingly, the survival rate was 100%. Leghissa *et al.* opted for a simultaneous implant placement, and the titanium mesh was covered by a non-resorbable barrier membrane to avoid non-osteogenic cells migration underneath. In this case, no graft was used and implants were characterized by a rough surface (16). A simultaneous approach was also adopted by Artzi *et al.*, preferring however DBBM as a graft material (19). A 8-9 months delayed implant placement was carried out by Pieri *et al.*, combining autogenous bone with DBBM graft in a 70:30 ratio (15). Finally, Corinaldesi preferred a mixed technique, in which both simultaneous and delayed approaches were performed depending on the patient, grafting particulated autogenous bone under the titanium mesh (21).

**Table 3:**
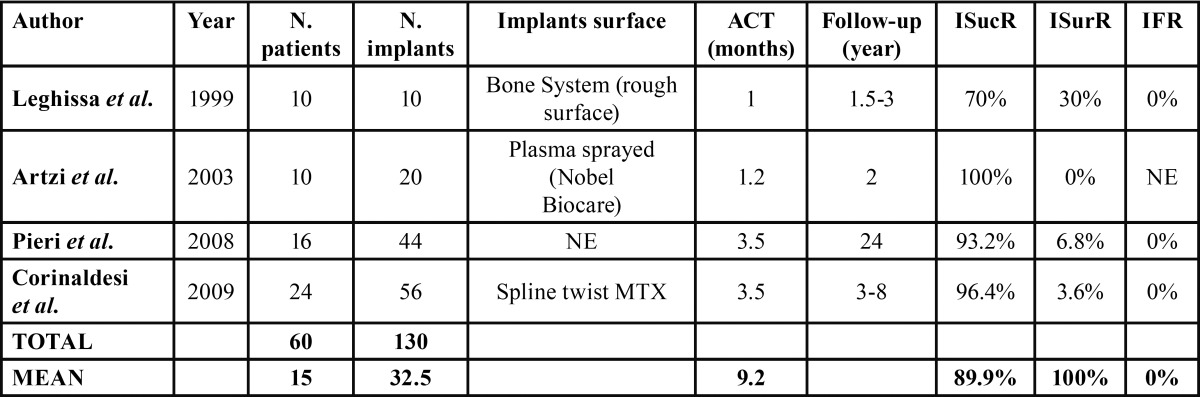
Evaluation of implant survival, success and failure rate.ACT: Abutment Connection Time; ISucR: Implants Success Rate; ISurR: Implants Survival Rate; IFR: Implants Failure Rate; NE: Not Evaluable.

## Discussion

From the analysis of the literature, few studies concerning alveolar ridge reconstruction with titanium mesh were published, above all no systematic or meta-analysis reviews were found. Hence, the aim of the present systematic review was to standardize the results reported in literature evaluating in detail three peculiar aspects: a) the obtained bone regeneration, b) the complications rate, c) the implants survival and success rate.

Inclusion and exclusion criteria were those typically proposed by most of the reviews. Particularly, studies involving patients presenting heavy smoker habits (>20 cigarettes/day) or previously affected by tumours or congenital malformations were dropped out,to avoid any presumable alteration of data regarding the healing and the volume of the graft. The sample was limited to bone defects caused by trauma, teeth extractions and periodontal disease. The topic was focused on the presence of the titanium mesh as a barrier device for ridge reconstruction in partial and complete edentulism, without considering the graft material or the operative protocol, including both simultaneous and delayed implant placement timing. In some protocols the mesh was used in combination with resorbable and non-resorbable membranes (16,17,22). This association was performed to prevent the migration of the overlying epithelial cells and fibroblasts in the regenerated bone, following the principles of guided tissue regeneration (GTR). In order to obtain bone regeneration, the role of the membranes as physical barriers towards the rapid turnover cells, such as endothelial cells and fibroblasts, is still a controversial topic. Indeed, in an experimental in vivo study, Salvatore *et al.* reported that reducing the pores size could accelerate the collagen and vascular tissue ingrowth (23). Differently, Chvapil *et al.* suggested that pores in excess of 100 μmwere required for the rapid penetration of highly vascular connective tissue, however small pores tend to become filled with more avascular tissue (24). In agreement, Taylor and Smith found that small poresize was inadequate for penetrations of capillaries (25). In a prospective histomorphometric study on adult hound dogs, Gutta *et al.* observed that macroporous membranes facilitated greater bone regeneration compared with microporous and resorbable membranes: the mean area of new bone formation in large and small meshes was 66.26 ± 13.78 mm2 and 52.82 ± 24.75 mm2 respectively, whereas in resorbable meshes was 46.76 ± 21.22 mm2, although differences among the groups were not statistically significant. Furthermore, better results in terms of soft tissue ingrowth were found with macroporous mesh compared to the other barriers.

Despite the incompleteness of data, the analysis of the bone regeneration obtained demonstrated satisfying results. Measurements related to the augmentation were reported in only 10 of the 17 articles. The mean vertical regeneration was 4,91mm while the mean horizontal augmentation was 4,36mm. Implants placement was possible in all cases. The review of the studies highlighted that the use of titanium mesh was an effective procedure both in vertical and horizontal defects reconstruction considering total and partial edentulism. Data emerged from the studies allowed to consider the increased bone volume, however there were not sufficient data to evaluate the resorption in the course of the time; therefore these two aspects were not comparable. In the analyzed studies, the limits of the comparison emerged from different aspects: different types of measures (linear versus volumetric), different measurements interval timing, the lack of comparison between desired bone volume and obtained bone volume. Despite the differences regarding the surgical protocols (simultaneous or delayed implant placement, with or without resorbable membrane association, different timing of mesh removal, different graft materials) results were similar. Consequently, was rather difficult to identify the most effective surgical technique associated with titanium mesh.

From the analysis of the complications, interesting considerations were found. The investigation was exclusively focused on membrane exposures, which was the most common complication in this surgical procedure. All the analysed studies high lighted the necessity to mobilize the flaps in order to obtain a primary wound closure without tensions, avoiding the premature exposure of the augmented area, jeopardizing the final outcome. The mean exposure rate was 16,1%. Comparing the present result with vertical bone augmentation performed with both resorbable and non-resorbable membranes, similar results were reported in a systematic review by Cordaro et al.: the mean complication rate, usually related to membrane exposure, was 13.1% when implants were placed simultaneously with GBR; in case of GBR and non-simultaneous implant placement, the mean complication rate was 6.95%, however only two studies were included for the delayed approach (26). A trend in favour of the reduction of the exposure rate of the titanium mesh was observed, therefore it could be hypothesized that both materials and techniques have been improved in the course of the years. Concerning the management of non-resorbable e/d-PTFE membranes in case of exposure, as a spontaneous healing of the dehiscence could never be observed, the removal of the device should be considered. In contrast, the mean values extracted in the present review illustrated that, when a mesh exposure occurred, only in 20% of the cases the membrane removal was necessary, while in the remaining cases, it was sufficient to treat the dehiscence with a topical application of chlorhexidine gel, avoiding the suprainfection of the site and a consequentbone loss. In case of an early exposure of the membrane, 3-4 weeks from the surgery, Corinaldesi *et al.* pointed out that the probability of mesh removal could be higher (21). Proussaefs *et al.* reported the most relevant exposure rate with 35,2% of complications, associated with the slightest bone regeneration values, consisted in an horizontal augmentation of 3,75mm and a vertical augmentation of 2,56mm (14). Consequently, a cause-and-effect relationship between membrane exposure and bone loss may be deduced, however it has to be considered that in all reports except one, implant placement could always be performed. The use of titanium mesh combined with the application of resorbable or non-resorbable membranes was reported by several authors to prevent the risk of dehiscences (16,22). Particularly, resorbable membranes may have a double function; favouring the creeping attachment of the soft tissues protecting at the same time the graft form the infiltration of epithelium cells. Nevertheless, it was authors opinion that a correct management and mobilization of the flaps was the prime determinant in the prevention of soft tissue dehiscences.

The success rate of implants placed in regenerated bone should not be confused with the survival rate. As before mentioned,in the present review Albrektsson *et al.* criteria were adopted,specifying that if the MBR was higher than 1,5mm after the first year, implant had to be considered survived. As a matter of fact, an implant could remain stable even if the regenerated bone has gone through resorption. For this reason, only four articles respected the Albrektsson *et al.* criteria (15,16,19,21), including a total sum of 60 patients and 130 implants with a mean success rate of 89,9%, a mean survival rate of 100% and a failure rate of 0%. These studies reported a remarkably high success rate, probably due to the limited number of cases treated. In a recent systematic review (27), all the studies except three, reported a success rate higher than 90% (range 90–100%), whereas the survival rate of implants reported in 6 studies, ranged from 93.75% to 100%. One study reported a survival rate lower than 99.2%. Both success and survival rate reported in the present review were in consistent with the current available literature (26,27).

The aim of the present review was to evaluate a specific bone regeneration method, focusing on the augmented obtained bone including implant survival, success and complication rate. In all of the analysed study the remarkable favourable characteristics of titanium were found. The vertical/horizontal bone regeneration obtained was always adequate in order to finalize the implant-prosthetic treatment. In the present review an elective operative protocol could not be evidenced, as the type of technique and the type of graft were not diriment in obtaining the final result. This type of bone regeneration technique seemed to be safer compared with the others because the onset of infection was rather rare. In the reviewed publications, the exposure rate resulted uncommon and comparable suggesting that this kind of complication is operator and not technique - dependent. The success and survival rate resulted noticeably elevated despite the randomized clinical trials sample was limited. Titanium mesh offered an excellent solution for alveolar ridge reconstruction. The clinical studies currently available in literature have shown the predictability of this technique in both lateral and vertical bone regeneration.
